# Molecular docking assisted biological functions and phytochemical screening of *Amaranthus lividus* L. extract

**DOI:** 10.1038/s41598-022-08421-8

**Published:** 2022-03-12

**Authors:** Burhan Durhan, Emine Yalçın, Kültiğin Çavuşoğlu, Ali Acar

**Affiliations:** 1 Institute of Science, Giresun University, Giresun, Turkey; 2 Department of Biology, Faculty of Science and Art, Giresun University, 28200 Giresun, Turkey; 3 Department of Medical Services and Techniques, Vocational School of Health Services, Giresun University, Giresun, Turkey

**Keywords:** Biological techniques, Biotechnology

## Abstract

In this study, the phytochemical content of *Amaranthus lividus* extract and its multi-biological activities were investigated. Total protein, phenol, flavonoid, saponin and condensed tannin contents were determined for phytochemical analysis. In addition, GC–MS and HPLC analyzes were carried out for the determination of the active components of the extract. In determining the multi-biological activities, radical scavenging, anti-mutagenic, anti-proliferative and anti-microbial activities of the extract were investigated. GC–MS analysis revealed that the leaf extract of *A. lividus* contains phytol and β-sitosterol as major compounds and the presence of gallic acid, caffeic acid, quercetin, vanillin and kaemferol compounds were determined with HPLC analysis. The radical scavenging effect of *A. lividus* extract was determined as 75.6% against 2,2-diphenyl-1-picrylhydrazyl and 85.2% against superoxide. In anti-bacterial studies, it was determined that *A.lividus* extract formed different inhibition zones against all tested bacteria. The highest inhibition zone was 14.3 ± 0.7 mm against *Bacillus subtilis*. In addition, the anti-microbial activity of the extract was demonstrated by molecular docking studies of the binding of gallic acid and phytol to aquaporin and arginase enzyme of bacteria, and the mechanism of anti-microbial activity was explained. *A. lividus* extract, which provided a 68.59–33.13% reduction in the formation of chromosomal aberrations such as unequal distribution of chromatin, micronucleus formation, fragment, sticky chromosome, bridge and vagrant chromosome, exhibited a strong anti-mutagenic effect. *A. lividus* extract has a reducing effect on the number of dividing cells and exhibits an anti-proliferative effect of 25.7% compared to the control group. The antiproliferative mechanism of action was investigated by molecular docking and it was determined that the gallic acid and phytol in the extract decreased proliferation by interacting with telomerase. As a result, *A.lividus* extract consumed as food is a potential natural anti-microbial, anti-oxidant, anti-mutagenic and anti-proliferative source with its rich phytochemical content.

## Introduction

Information on the therapeutic properties of plants used for medicinal purposes by humans until the middle of the nineteenth century date back to ancient civilizations. Inadequacies in the treatment of various diseases such as AIDS, cancer, hepatitis, diabetes, mental disorders and allergies have led to the search for new phytochemicals and natural products that can be used as drugs^1,2^. Various extracts of different parts of plants are widely used in medical applications, folk remedies and perfumes, as well as various additives such as food flavorings and preservatives. Medicinal plants are an important biological resource for traditional medicine systems, food supplements, pharmaceutical intermediates, nutraceuticals, raw and synthetic drugs. With the developments in phytochemical techniques, various active ingredients of many medicinal plants have been isolated and started to be used as valuable drugs in modern medical systems. Alkaloids, tannins, flavonoids and phenolic compounds are the most important of these bioactive compounds. Considering the rich plant diversity in the world, studies on existing bioactive natural products are not yet at the desired level^3,4^. In this study, phytochemical content and multi-biological properties of *A. lividu*s L. extracts were investigated. *Amaranthus* plants (Amaranthaceae) can spread all over the world and can grow in various climatic conditions. Although many species are belonging to the genus *Amaranthus* in the world, in Turkey, nine species including *A. albus*, *A. chlorostachys*, *A. blitoides*, *A. deflexus*, *A. lividus*, *A. graecizans*, *A. retroflexus*, *A. patulus* and *A. viridis* are reported^5,6^. *Amaranthus* plants are used worldwide as a food source. Amaranth oil, found in various tissues of the plant, contains high levels of unsaturated fat (about 70% oleic acid) and is a rich source of tocotrienol and squalene. Leaves of *Amaranthus* are also rich in minerals such as protein, carotenoids, vitamin C, calcium, iron, zinc, magnesium and phosphorus. In the Black Sea region, especially in Giresun, the leaves of *A. lividus* are consumed as a vegetable^7,8^. *A. lividus* is a plant belonging to the family Amaranthaceae and the genus *Amaranthus*. A*. lividus* extract has also been reported to be used in the treatment of mouth and throat ulcers, diarrhea, dysentery, ulcers and intestinal bleeding^9^. *A. lividus* is also used to relieve coughs, colds and to control excessive bile secretion. *A. lividus* root extract is also known to be used against stomach problems, menorrhagia, boils, burns and nausea. It has been reported in the literature that *A. lividus*, known for its anti-pyretic, appetizing and diuretic properties, is also used in the treatment of hallucinations, leprosy, and eczema^10^. The protective and therapeutic properties of *A. lividus* against various diseases are due to the active components in its structure.

In this study, phytochemical analysis of *A. lividus* extract was investigated with phenolic and flavonoid compounds, condensed tannin and saponin contents. Especially anti-oxidant compounds have an important role in the biological activity of a plant. Antioxidants are the focus of many studies day by day due to their ability to remove the negative effects of free radicals. In this context, phenolic and flavonoid compounds in *A. lividus* extract, tannin and saponin content, which exhibit strong anti-microbial activity, were also investigated, and GC–MS and HPLC analyzes were carried out to detect bioactive compounds. The biological and pharmacological activities of *A. lividus* are due to the active components contained in the plant. Therefore, active compounds were determined by phytochemical analysis and advanced chemical techniques and associated with the biological functions of *A. lividus*. The multi-biological effects of *A. lividus* extract were investigated with anti-microbial, anti-proliferative, anti-mutagenic, radical scavenging and anti-diabetic activities. Today, all organisms, including humans, are exposed to radiation, industrial wastewater, food additives and pesticides. As a result of all this exposure, new diseases appear or an increase in the frequency of existing diseases is observed. Consumption of natural anti-mutagenic, anti-microbial, anti-oxidant or anti-diabetic products in the daily diet reduces the risk of developing these diseases. Anti-mutagenic and anti-proliferative effects are very important in preventing cancer formation and proliferation of cancer cells, and products with this feature are defined as anti-cancer agents^11^. Although there are many studies in the literature investigating the radical removal, anti-proliferative and anti-mutagenic effects of plant tissues, the high diversity of plants in the world and Turkey makes these studies insufficient. Although many studies are investigating the content of *A. lividus* extracts collected from different ecological environments in the literature, there is no study evaluating the biological/pharmacological effects associated with the phytochemical content together.

In this study, a detailed phytochemical analysis was performed with total phenol, total flavonoid, condensed tannin and total saponin contents, HPLC, GC–MS analyses of *A. lividus* extracts. In addition, the radical scavenging, anti-mutagenic, anti-proliferative and anti-microbial effects of the extract were investigated and associated with the phytochemical content. In addition, the anti-microbial and anti-proliferative activities of the extract were demonstrated by molecular docking studies.

## Materials and methods

### Sample preparation and extraction

*A. lividus* samples were collected in Giresun, August 2020 and its identification was made in the Botany Department of Giresun University, a sample was archived in the herbarium. Experimental research and field studies on plants, including the collection of plant material, comply with relevant institutional, national, and international guidelines and legislation. *A. lividus* leaves were dried and powdered under sterile conditions in the laboratory environment. Three different solvents were used in the extraction stage and the effect of each solvent was tested by investigating the bioactive component ratios in the liquid extract. Leaf sample (10 g) was mixed with 100 mL of ethanol, methanol and water solvents and left for extraction in a shaking incubator at room temperature for 2 h. At the end of the incubation period, the solid particles were filtered and the resulting filtrate was centrifuged at 10,000 rpm for 10 min. After centrifugation, the supernatant was evaporated and the obtained pellet was used for further analysis.

### Extraction efficiency

The amount of extractable substance (g) obtained with each solvent was used to evaluate the extraction efficiency. For this purpose, solid particles obtained after extraction with different solvents were weighed with precision balance. In the comparison of the extraction efficiency of the solvents, besides the amount of extracted substance, the total phenolic substance and flavonoid contents were also investigated.

The phenolic content of *A. lividus* extract was determined by the Folin-Ciocaltaeu method. Gallic acid was used as the standard substance. A mixture containing 0.5 mL sample, Folin-Ciocaltaeu reagent (2.5 mL, 10%) and 7.5 mL Na_2_CO_3_ (20%) was prepared for experimental procedures. This mixture was left in the dark at room temperature for 2 h and determined spectrophotometrically at 750 nm. The total phenolic substance concentration was evaluated as gallic acid equivalent (mgGAE/100 mg dry weight). For flavonoid determination, 10 mL of sample and 1 mL of sodium nitrite were mixed and kept for 6 min. Then 1 mL of Al(NO_3_)_3_ was added and the final mixture was incubated for 6 min and the total volume was made up to 25 mL with dH_2_O. The absorbance of the solution was determined spectrophotometrically at 510 nm after 15 min incubation. Quercetin was used as the standard and flavonoid amounts were expressed as mg QE/100 mg dry weight^12^.

### Condensed tannin and total saponin

Determination of condensed tannin based on the principle of precipitation with formaldehyde was made according to the method suggested by Vermerris and Nicholson^13^. Condensed tannin concentration was determined using total phenolic and residual phenol concentrations. Condensed tannin content is expressed as gallic acid equivalents (mg GAE/g extract).

To determine the total saponin content of the *A. lividus* extract, a mixture containing 0.25 mL of sample, 0.25 mL of vanillin and 2 mL of sulfuric acid was prepared and incubated at 60 °C for 15 min. The absorbance of the cooled mixture was read at 538 nm. Quillaja was used as a standard and the total saponin content was calculated as mg QAE/g extract^14^.

### GC–MS analysis

GC–MS analysis was performed on the Perkin Elmer system, using a silica capillary column. Helium (99.9%) was used as the carrier gas, with a constant flow of 1 mL/min and an injection volume of 0.5 EI. An isothermal program was used at 110 °C for 2 min, in 10 °C/min increments to 200 °C, then from 5 °C/min to 280 °C and 9 min at 280 °C. Mass spectra were taken at 70 eV, with a scanning interval of 0.5 s^15^.

### HPLC analysis

The dried crude extract was dissolved in 100 mL of mobile phase and filtered through a 0.45 mm membrane filter (Millipore) and injected into HPLC. C-18 reversed phase column was used in the analysis. Phase A was determined as water-acetic acid (25:1 v/v) and phase B was methanol. Phase B was increased to 50% in 4 min and a gradient profile was created to increase to 80% in 10 min. Flow rate was determined as 1.0 mL/min and reading was taken at 280 nm wavelength. Gallic acid, vanillin, caffeic acid, rutin kaemferol, ferulic acid and quercetin were used as standards. The phenolic acids present in each sample were identified by comparing the retention times (Rt) of the standards^16^.

### Radical scavenging activity

The radical scavenging effect of *A. lividus* extract was tested against superoxide and 2,2-diphenyl-1-picrylhydrazy (DPPH) radicals. Superoxide radical scavenging activity and DPPH removal activity was carried out according to the method suggested by Gündüz et al.^12^. BHT was used as a standard in both radical scavenging activities.

### Anti-bacterial and anti-fungal activity

The disk diffusion method was used to determine the anti-microbial activities of *A. lividus* extract. Anti-bacterial activity of the extracts was studied against gram-negative (*Escherichia coli* ATCC25922, *Salmonella typhimurium* ATCC14028, *Pseudomonas aeruginosa* ATCC27853, *Klebsiella pneumoniae* ATCC11296) and gram-positive (*Streptococcus pyogenes* ATCC19615, *Bacillus subtilis* ATCC35021, *Staphylococcus aureus* ATCC25923, *Staphylococcus epidermidis* ATCC12228) bacteria. *Candida krusei* ATCC6258 and *Candidia albicans* ATCC102316 species were used to determine the anti-fungal activity. For the disc diffusion method, extract-impregnated discs (20 µL/disc) were placed in petri dishes inoculated with microorganisms and incubated for 24 h at 37 °C for bacteria and 27 °C for fungi. Inhibition zones formed on the medium after incubation were evaluated as mm^17^. Nystatine and Amikacin antibiotics were used as standard.

### Molecular docking

To elucidate the potential mechanism of anti-microbial and anti-proliferative activity, molecular docking studies of the bacterial components and the active ingredients of extract were carried out. So, molecular docking was performed to analyze the potential interactions of phytol and gallic acid with aquaporin-z, arginase and telomerase. The 3D crystal structure of the aquaporin-z (PDB ID: 1RC2)^18^ and arginase (PDB ID: 2CEV)^19^ and telomerase (5CQG)^20^ molecules were obtained from the protein data bank. The 3D structures of gallic acid (PubChem CID: 370) and phytol (PubChem CID: 5280435) were retrieved from the PubChem. Proteins were prepared using Biovia Discovery Studio 2020 Client for docking. For the docking process, in crystallographic structures chain A was used structures. In the preparation process, the active site was determined, after removing the water molecules and co-crystal ligands, polar hydrogen was added. Energy minimization was done with Gromos 43B1 using Swiss-PdbViewer^21^ (v.4.1.0) software for proteins, ligand energy minimization was done with the uff-force field using Open Babel v.2.4.0 software^22^. The regions of the amino acids interacting with the ligands present in the protein files were determined as active regions, and a grid box covering these regions was created. For 1RC2, the grid box was created with size 60 × 60 × 54 xyz points, grid spacing of 0.375 Å and grid center of x, y and z dimensions of 70.941, 15.337 and − 39.011, respectively. For 2CEV, the grid box was set at 60 × 98 × 66 xyz points with grid spacing of 0.375 Å and grid center was designated at dimensions (x, y and z): 18.019, 96.719 and 51.02. Then docking was performed using Autodock 4.2.6 software^23^ based on Lamarckian genetic algorithm (LGA) and the LGA was run for 10 runs with an initial population size of 150 individuals for both enzymes and DNA. The docking analysis and 3D visualizations were performed with Biovia Discovery Studio 2020 Client.

### Anti-proliferative activity

The anti-proliferative effect of *A. lividus* extracts was determined by the *Allium* test using mitotic index (MI) percentages. For this purpose, three groups were formed and *Allium* bulbs were germinated under sterile conditions for 72 h. The control group, positive control and application group were germinated with dH_2_O, 75 mg/L Glyphosate and 75 mg/L *A. lividus* extract (Group III), respectively. After incubation, meristematic slides were prepared and mitotic cells were examined. 5 slides were prepared for each group, 1.000 cells from each slide, a total of 5000 cells were counted and MI percentages were calculated using Eq. (1)^24^.

1 $$ {\text{Mitotic index }}\left( \% \right) \, = {\text{ Number of dividing cells}}/{\text{total number of cells }} \times { 1}00. $$

Cells in prophase, metaphase, anaphase and telophase were taken as a basis in determining the number of dividing cells.

### Anti-mutagenic activity

The anti-mutagenic activity of *A. lividus* extract was determined using the frequency of chromosomal abnormalities (CAs) detected in the *Allium* test. For this purpose, four groups were formed and *Allium* bulbs were germinated under sterile conditions for 72 h. Group I was treated with tap water and accepted as the negative control. Group II was treated with NaN_3_, a potent mutagen, and was considered a positive control. Groups III and IV were treated with extract only and NaN_3_ + extract, respectively. It was determined whether *A. lividus* extract was mutagenic or not with the data obtained only in the extract application group. In the group in which both the extract and the positive mutagen were applied together, the decrease in the mutagenicity induced by the positive mutagen was examined and the anti-mutagenic effect of extract was determined^25^. The anti-mutagenic activity was calculated using Eqs. (2) and (3).

2 $$ \% {\text{ CA}} = {\text{ A}}/{\text{B }} \times {1}00. $$

A: number of cells with abnormal chromosomes, B: total counted cells

3 $$ \% {\text{ Anti-mutagenicity}}  = \, \left[ {\left( {{\text{a}} - {\text{b}}} \right)/\left( {{\text{a}} - {\text{c}}} \right)} \right] \times { 1}00. $$

a: %CA of NaN_3_ applied group, b: %CA of plant extract + NaN_3_ applied group, c: %CA of the control group.

### Statistical analysis

Analyzes were performed with the “IBM SPSS Statistics 22” package program and the data were given as mean standard deviation (SD). Statistical significance between the means was determined by Duncan’s test and One-way ANOVA, and a p value of < 0.05 was considered statistically significant.

## Results and discussion

In this study, phytochemical analysis and multi-biological functions of *A. lividus* leaf extract were investigated. Total phenolic, total flavonoid, saponin and condensed tannin contents were determined in phytochemical analyzes and also GC–MS and HPLC analyzes were performed. The biological functions of the extract were determined by testing its free radical scavenging, anti-bacterial, anti-fungal, anti-mutagenic and anti-proliferative activities. Each biological function was evaluated concerning the phytochemical content and compared with the literature examples. The mechanisms of anti-bacterial and antiproliferative activity of the extract were also interpreted by molecular docking studies.

### Extraction efficiency, phenolic and flavonoid content

**Figure 1:**
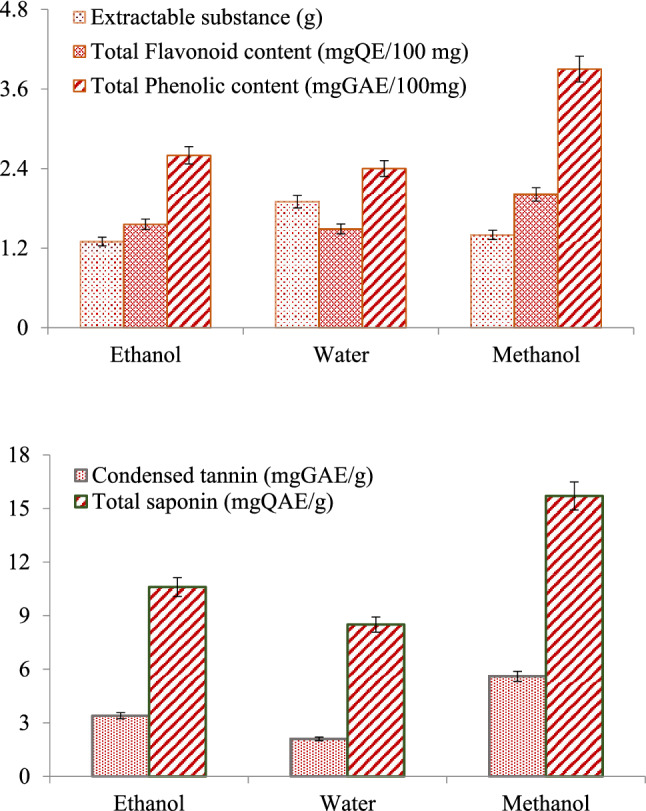
The amount of extractable substance, total phenolic, flavonoid, condensed tannin and saponin contents of *A. lividus* extract in different solvents. Each analysis was repeated three times and average values are given.

*A. lividus* leaves were extracted in different solvents and the extraction efficiency was determined by investigating extracted substance, the phenolic and flavonoid content in each solvent. The total phenolic and flavonoid contents obtained by extraction in different solvents and the amount of extracted substance are given in Fig. 1. The highest extractable yield was obtained with water extraction, followed by methanol and ethanol, respectively. After extraction with water, methanol and ethanol, 1.9 g, 1.4 g and 1.3 g solids were obtained, respectively. The role of phenolic and flavonoid compounds in the antioxidant properties of herbal extracts is quite high. Although the amount of extract obtained with water is high, methanol and ethanol solvents were found to be more effective in the extraction of phenolic and flavonoid substances. A medium level of phenolic content was determined in *A. lividus* leaf extract and the highest phenolic content was found as 3.9 mgGAE/100 mg in the extraction with methanol. In terms of phenolic content, the performances of the solvents showed an order of methanol > ethanol > water. Flavonoid compounds obtained from *A. lividus* leaf extract were found as 1.49, 2.01 and 1.56 mgQE/100 mg for water, methanol and ethanol solvents, respectively. In terms of flavonoid content, the efficiency of the solvents were ranked as methanol > ethanol > water, but the performances of ethanol and water solvents were similar.

Many studies have shown that methanol and ethanol are effective solvents, especially for the extraction of phenolic compounds^26^. The polarity of the solvent used in the extraction of phenolic and flavonoid compounds and the solubility of each compound are very important factors. Differences in the polarity of the solvents used in the extraction may also cause variation in the solubility of bioactive compounds. The high efficiency of polar solvents in the extraction of plant tissues has been demonstrated by many studies. Plant tissues contain polar compounds with high solubility in polar solvents such as water, methanol and ethanol. In this study, the higher yield of phenolics and flavonoids obtained in the methanolic extract may be associated with the higher solubility of these compounds in methanol. Similarly, Truong et al.^27^ reported that the methanolic extract of *Severinia buxifolia*, which they extracted with different solvents, exhibited higher phytochemical content, anti-oxidant and anti-inflammatory activity compared to all other extracts tested. Another important result obtained from our study is that the phenolic content detected in the leaf extract of *A. lividus* is higher than the flavonoids. It has been reported in the literature that various phytochemical compounds were determined in *A. lividus* extracts. Ozsoy et al.^5^ extracted the leaf and flower tissues of *A. lividus* and reported that the highest phenolic content was obtained by ethyl acetate extraction and they found a phenolic content of 22.8 mg GAE/g. Nehal et al.^10^ identified flavonoid content at the range of 4.81–5.04 RE/mg under different extraction conditions in *A. lividus* leaves.

### Condensed tannin and total saponin content

Condensed tannin and saponin contents of the *A. lividus* leaf extract obtained in different solvents are given in Fig. 1. While the highest saponin and tannin contents were obtained in the extracts obtained with methanol, similar levels of each content were obtained in the ethanol and water extracts. It was determined that methanol, ethanol and water extracts contained 15.7 mg QAE/g, 10.6 mg QAE/g and 8.5 mg QAE/g saponin, respectively. The condensed tannin content was determined as 5.6 mg GAE/g in the methanol extract at a lower level compared to the saponin. Saponins are a phytochemical found structurally in many species of both wild and arable plants. It is also known that saponins protect plants against microorganisms, fungus and insect attacks. Saponins have hypoglycemic activity, cholesterol-lowering effect, anti-inflammatory and anti-oxidant activity^28^. The wide varieties of biological activities of saponins enable them to exhibit protective properties against many diseases and toxicities. The intense saponin content detected in the extract indicates that consumption of *A. lividus* in the daily diet will reduce risks such as hyperglycemia, inflammation and hypercholesterolemia.

*A. lividus* leaves, which contain less condensed tannin compared to saponins, gain various biological properties due to the biological activities of tannin molecules. Condensed tannins, also known as proanthocyanidins, are composed of numerous flavonoid units linked by carbon–carbon bonds. In addition to their high anti-oxidant properties, condensed tannins have anti-inflammatory, anti-asthmatic, anti-cancer, anti-viral, anti-carcinogenic, anti-allergic, anti-microbial, anti-hypertension and preventive properties against cardiovascular risks. Wound healing, reducing insulin resistance, protection against drug toxicity, and cholesterol-lowering effects of condensed tannins are also reported in the literature^29^. Saponin and condensed tannins, which have intense biological activity, ensure that the leaves of *A. lividus* exhibit significant effects.

### GC–MS and HPLC analysis

**Figure 2:**
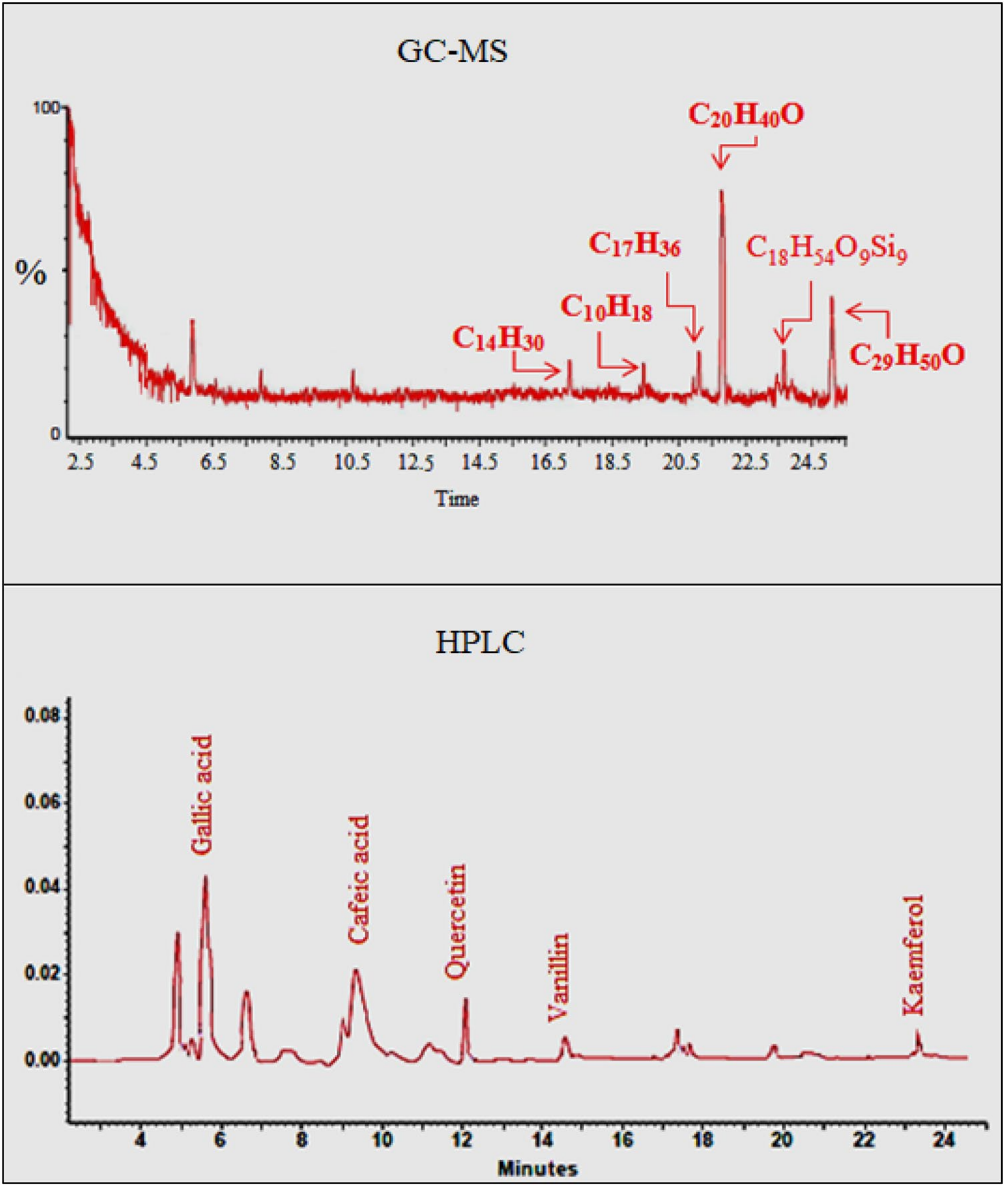
GC–MS and HPLC spectrum of *A. lividus* extract. C_14_H_30_: tetradecane, C_10_H_20_: cyclodecane, C_17_H_36_: heptadecane, C_20_H_40_O: phytol, C_18_H_54_O_9_Si_9_: oktademetilcyclolononasiloxane, C_29_H_50_O: β-cytosterol.

The GC–MS spectrum of *A. lividus* extract is given in Fig. 2 and the presence of various bioactive compounds was determined. The compounds detected in extract were ranked as phytol > β-sitosterol > octademethylcyclononasiloxane > heptadecane > tetradecane > cyclodecane according to their presence rates. Among the active ingredients, phytol has a high rate as the major component. Phytol has anti-cancer, anti-oxidant, anti-inflammatory, anti-tumor, anti-microbial, diuretic properties and is frequently used in vaccine formulations^30^. Phytol has strong anti-oxidant activity due to the allylic structure of the alcohol group. The antioxidant activity of phytol occurs by the reaction of hydrogen atoms with free radicals, resulting in the conversion of free radicals to less reactive species^31^. Phytol, which has a lipophilic character, also exhibits anti-bacterial properties by passing through bacterial cell membranes and damaging macromolecules such as proteins, lipids and DNA within the cell^32^. β-sitosterol, one of the other major compounds detected in GC–MS, plays an important role in the regulation of fatty acid chains in the cell membrane. The anti-microbial and anti-tumor properties of β-sitosterol are also reported^33^. Hydrocarbons with high molecular weight such as heptadecane, tetradecane and cyclodecane detected in the extract content exhibit anti-microbial activity by showing an inhibitory effect on microorganisms^34^. Biological activities of all bioactive components detected by GC–MS analysis ensure that *A. lividus* leaves also exhibit multi-biological activity. In the literature, the phytochemical contents of *Amaranthus* species collected from different ecological environments were investigated by GC–MS analysis. Nehal et al.^10^ reported that *A. lividus* leaf and stem extract contains high levels of β-sitosterol and phytols as a result of GC–MS analysis, and minor compounds such as ergost-5-en-3-ol, (3, β), hexadecanoic acid and tetradecane. Paranthaman et al.^35^ determined that the extracts of *Amaranth caudatus* contain high levels of phytol, while at lower rates they contain many minor compounds such as pseudoephedrinei, 1,2,4-butanetriol, N-ethyl-N′-nitroguanidine.

HPLC spectrum of *A.lividus* extract is given in Fig. 2 and the presence of various phenolic compounds in the extract was determined. Gallic acid, vanillin, caffeic acid, rutin, kaemferol, ferulic acid and quercetin were used as standards, and the presence of gallic acid, caffeic acid, quercetin, vanillin and kaemferol compounds were determined in the *A. lividus* extract among the tested standards. The rates of gallic acid and caffeic acid in the extract are higher than the other active ingredients, and therefore these two compounds were identified as the major compounds in the extract, while quercetin, vanillin and kaemferol were defined as minor compounds. Gallic acid (3,4,5-trihydroxybenzoic acid), a low molecular weight triphenolic compound, is a powerful anti-oxidant, anti-inflammatory, anti-tumor, anti-diabetic and anti-obesity agent^36,37^. Caffeic acid, one of the major compounds in the extract, has many pharmacological properties such as anti-inflammatory, anti-cancer and anti-viral. It is also an antioxidant that can reduce oxidative stress caused by free radicals^38^. Vanillin and kaemferol detected as minor compounds in the extract have a wide variety of pharmacological effects, including anti-oxidant, anti-microbial, anti-cancer, neuroprotective and anti-diabetic activities^39,40^. Quercetin, a flavonoid glycoside detected in the extract by HPLC analysis, is a compound with anti-cancer, anti-tumor, anti-ulcer, anti-allergy, anti-viral, anti-inflammatory, anti-diabetic, anti-hypertensive and immunomodulatory activities^41^. The cumulative effects of active metabolites such as gallic acid, caffeic acid, quercetin, vanillin and kaemferol provide a high biological and pharmacological effect to *A. lividus* leaf extract. Similarly, Paranthaman et al.^35^ found that *Amaranthus caudatus* extract contains intense amounts of gallic acid and rutin, and lower amounts of caffeic acid, ferulic acid and quercetin by HPLC analysis.

### Radical scavenging activity

**Figure 3:**
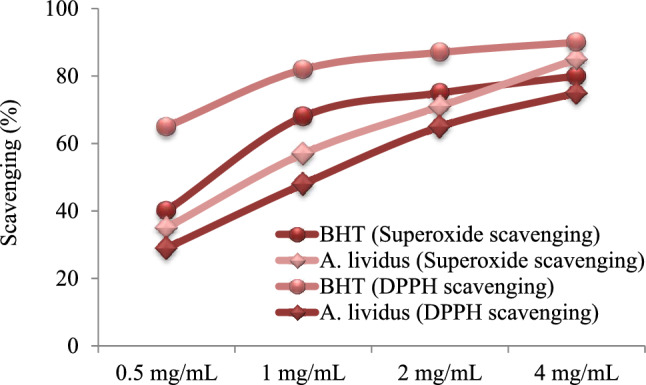
Radical scavenging activity of *A. lividus* leaf extract.

The radical scavenging activity of *A. lividus* leaf extract, which has rich phytochemical content, was tested against DPPH and superoxide radicals, and the results are given in Fig. 3. The radical scavenging activities of *A. lividus* extract and standard BHT increased with the increase in dose and the highest scavenging activity of *A. lividus* extract was obtained at a dose of 4 mg/mL. DPPH removal activity of 4 mg/mL BHT and *A.lividus* extract was determined as 90.1% and 75.6%, respectively. Superoxide removal activity was determined as 80.9% for BHT and 85.2% for *A. lividus* extract. *A. lividus* extract showed a higher activity in removing superoxide, which is a dangerous free radical in organisms, compared to BHT, a synthetic antioxidant. Superoxide formed during the reduction of oxygen in cells has radical properties and is an important reactive oxygen product. The superoxide radical reacts with organic substrates in the cell, leading to the formation of more intermediates that cause oxidative damage^42^. The superoxide radical formed in the cell under normal conditions can be neutralized by the endogenous anti-oxidants. However, the formation of superoxide at a higher level that can disrupt oxidant/antioxidant balance causes oxidative damage in the cell, and therefore exogenous anti-oxidant supplementation gains importance. The determination of superoxide removal activities of plants with high anti-oxidant content also comes to the fore. DPPH is a free radical and anti-oxidants react with DPPH by providing an electron or hydrogen atom and form the reduced product DPPH-H. Spectrophotometric measurement of this reduction reaction is used to determine the anti-oxidant activity of medicinal plants, fruits or other biological substrates^43^.

The high superoxide and DPPH scavenging activity of *A. lividus* extract show its high anti-oxidant activity. This activity is related to the phytochemical content of *A. lividus* extract. The saponins in *A. lividus* contribute to the radical scavenging activity with their proton supply capacity, free radical inhibition and primary anti-oxidant functions^44^. Tannins, like saponins, contribute to the radical scavenging activity by providing hydrogen atoms or electrons^45^. Phytol, the major compound, also contributes to the radical scavenging activity. Phytol exhibits good anti-oxidant activity due to the allylic structure of the alcohol group. The anti-oxidant activity of phytol is realized by the mechanism of reaction with free radicals and conversion to less reactive species^31^. Phenolic compounds detected in the extract by HPLC analysis also have an intense contribution to the radical scavenging activity. Gallic acid, the major compound in the extract, is a versatile radical scavenger that provides rapid neutralization of a wide variety of reactive oxygen species via electron transfer^46^. Bioactive compounds detected in all phytochemical analyzes carried out within the scope of this study provide the formation of radical scavenging activity of *A. lividus* leaf extract, and major compounds such as gallic acid, caffeic acid and phytol contribute to this effect being stronger. Natural antioxidant production in organisms decreases due to increasing age and exposure to intense oxidative stress. It is an important alternative to herbal antioxidants to close this gap. Herbal antioxidants play a role in preventing abnormal cell proliferation and protecting cells damaged by oxidation. With its intense antioxidant content and high radical scavenging activity, *A. lividus* protects macromolecules against oxidation and ensures the continuation of cell viability. In the literature, the radical scavenging activity of *A. lividus* samples grown under different ecological conditions was tested against various radical species and different results were obtained. Nehal et al.^10^ reported that *A. lividus* leaf extract, distributed in India, exhibited a DPPH removal activity of approximately 80% at a dose of 5 mg/mL. Ozsoy et al.^5^ determined that *A. lividus* samples grown in Bartın (Turkey) exhibited 89.9% DPPH removal activity at 40 mg/mL concentration and 92.8% hydroxyl radical removal activity at 20 mg/mL concentration.

### Anti-bacterial and anti-fungal activity

The anti-bacterial and anti-fungal activities of *A. lividus* extract are given in Table 1. Different inhibition zones were formed against all tested bacterial species. The highest inhibition zone was 14.3 ± 0.7 mm against *B. subtilis*, and the lowest zone was 7.7 ± 0.5 mm against *P. aeruginosa*. The fact that *A. lividus* extract forms an inhibition zone against all tested gram-negative and gram-positive bacteria indicates that it has a broad spectrum. However, *A. lividus* extract generally showed higher anti-bacterial activity against gram positives when compared to gram-negatives. This selectivity against bacteria is due to the structural difference between gram-positive and gram-negative bacteria. In gram-negative bacteria, the inner and outer membrane surrounding the cell is the main cause of resistance to anti-microbial compounds. The outer membrane of gram-negative bacteria provides resistance to bactericidal effects by preventing many compounds from entering the cell. Since this selective outer membrane is absent in gram-positive bacteria, anti-microbial compounds show higher efficacy^47^. *A. lividus* extract formed a similar inhibition zone against *C. krusei* and *C. albicans*. When the anti-fungal and anti-bacterial activities of *A. lividus* extract were compared, it was observed that a higher inhibition zone was formed against bacteria. This indicates that the extract is more effective against bacteria compared to fungi. This result is closely related to the cell wall of fungi, especially rich in chitin and ergosterol. It is known that *Candida* species increase the development of resistance by increasing chitin levels in the cell wall in the presence of anti-microbial agents^48^. The anti-bacterial and anti-fungal activity of *A. lividus* extract is closely related to the active ingredients it contains. Phytol, which is determined as a major compound and has a lipophilic character, can easily pass through the cell membrane in bacteria, causing disruption of membrane integrity, loss of basic cellular components and oxidative damage by damaging macromolecules such as proteins, lipids and DNA^32^. Gallic acid detected in the extract inhibits efflux pumps, folate synthesis and arginase activity in bacteria^49^. The interaction of gallic acid and phytol with bacterial components was also demonstrated by molecular docking study and potential bindings were also revealed in this study. Vanillin, one of the minor compounds, disrupts the membrane integrity in bacteria, causes intracellular K ions to leak out of the cell and changes the intracellular pH homeostasis^50^. The active compounds detected by GC–MS and HPLC have cidal effects on bacteria and fungi by different mechanisms, which enables *A. lividus* to exhibit anti-bacterial and anti-fungal activity.

**Table 1:** Anti-bacterial and anti-fungal activity of *A. lividus* extract.

	Microorganism	Inhibition zone of extract (mm)	Inhibition zone of amikacin (mm)	Inhibition zone of nystatin (mm)
Gram-negative	*K. pneumoniae*	8.5 ± 0.7	17.1 ± 0.8	ND
	*E. coli*	9.1 ± 0.3	18.1 ± 0.4	ND
	*P. aeruginosa*	7.7 ± 0.5	19.6 ± 0.4	ND
	*S. typhimurium*	10.1 ± 0.8	22.8 ± 0.3	ND
Gram-positive	*B. subtilis*	14.3 ± 0.7	20.9 ± 0.5	ND
	*S. pyogenes*	12.6 ± 0.5	17.1 ± 0.6	ND
	*S. aureus*	9.9 ± 0.6	17.8 ± 0.7	ND
	*S. epidermidis*	11.9 ± 0.4	21.3 ± 0.9	ND
Fungi	*C. krusei*	7.5 ± 0.6	ND	19.7 ± 0.4
	*C. albicans*	7.1 ± 0.4	ND	18.6 ± 0.9

*ND* not determined.

*Nystatin (30 µg), Amikacin (30 μg/mL).

In the literature, the anti-microbial activity of *Amaranthus* species was tested and different degrees of inhibition zones were reported. Nehal et al.^10^ investigated the anti-bacterial activity of *A. lividus* leaf and stem extract and reported that the highest effect was obtained against *B. subtilis* with an inhibition zone of 12.7 mm. They determined that an inhibition zone was not formed against *S. aureus* and *P. aeruginosa*. Mayio et al.^51^ observed that *A. hybridus*, *A. spinosus* and *A. caudatus* species had different degrees of anti-bacterial activity and were ineffective against *Candida albicans*. They stated that the anti-bacterial activity of the *Amaranthus* species was related to active pharmacological compounds such as flavonoids, steroids and terpenoids.

### Molecular docking for anti-bacterial activity

**Figure 4:**
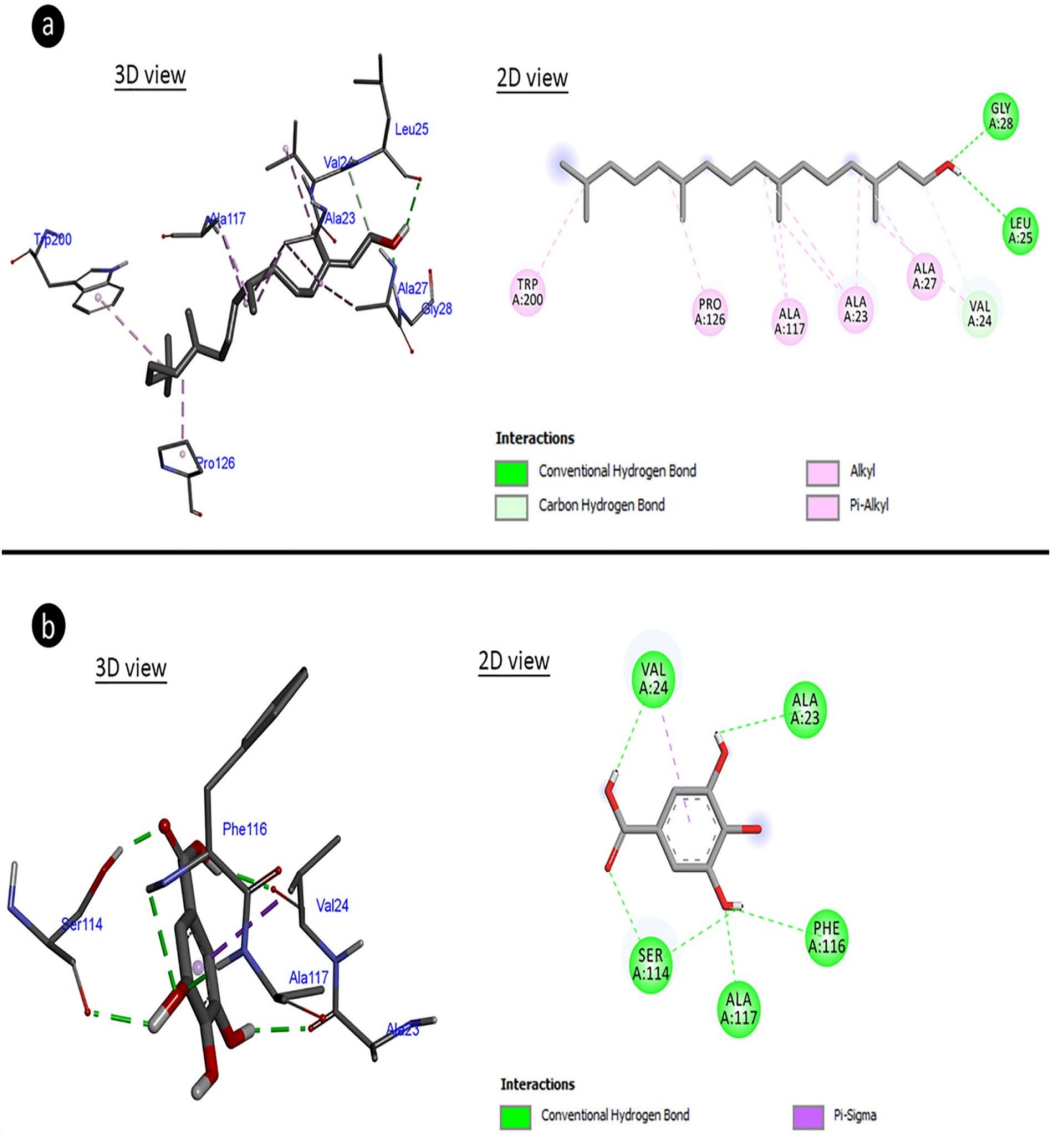
Potential molecular interactions, binding affinities and potential molecular interactions of gallic acid and phytol with aquaporin. (**a**) Aquaporin-phytol complex, (**b**) aquaporin-gallic acid complex.

**Figure 5:**
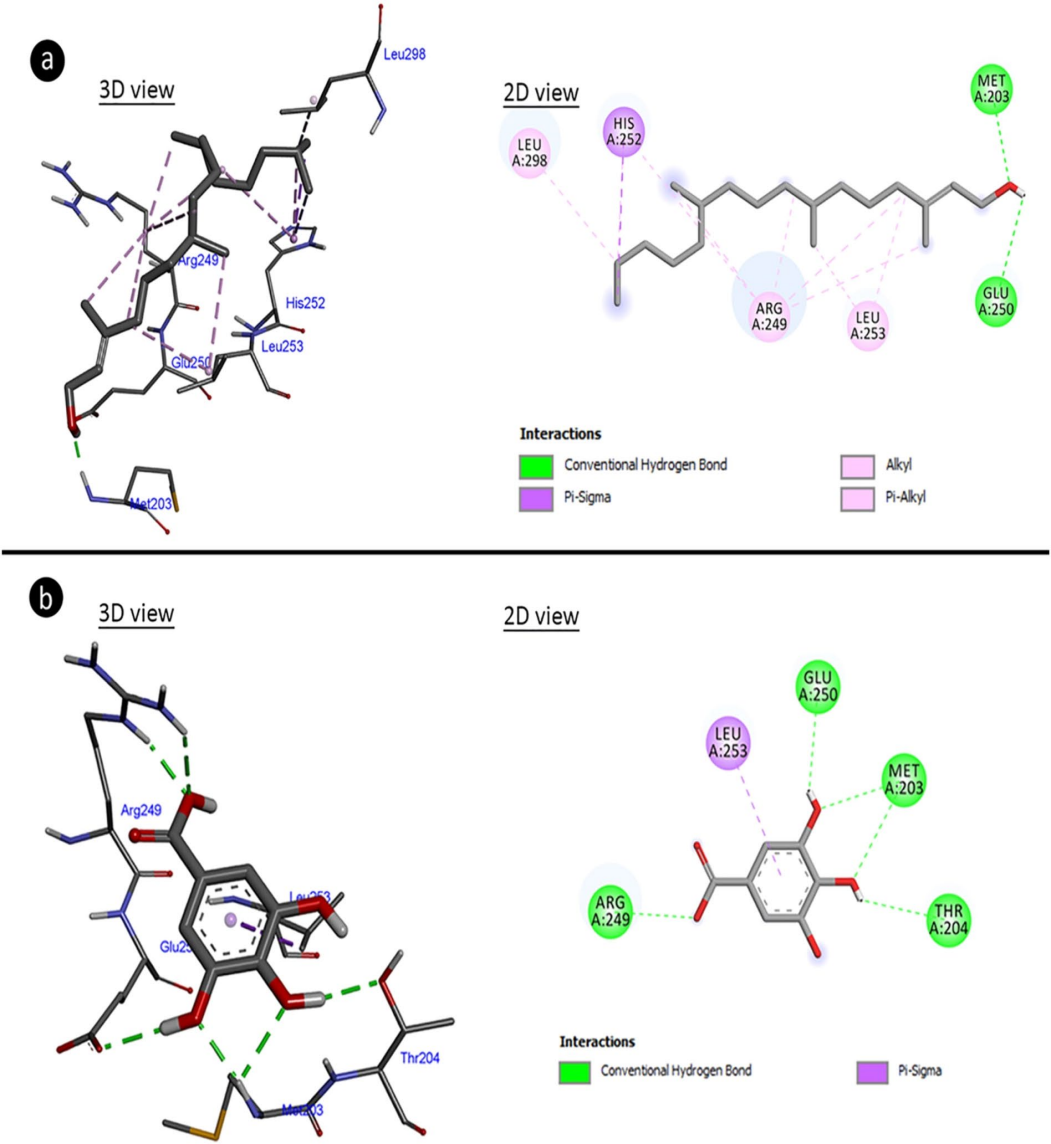
Potential molecular interactions, binding affinities and potential molecular interactions of gallic acid and phytol with arginase. (**a**) Arginase-phytol complex, (**b**) arginase-gallic acid complex.

In order to understand the mechanism of antimicrobial activity of the extract, molecular docking of active ingredients in extract with bacterial proteins was performed. The major components, which are intensely found in an extract, have an important role in the formation of biological activity compared to the minor components. For this reason, gallic acid and phytol, which are the major compounds detected in *A. lividus* extract by HPLC and GC–MS analyses, respectively, were used in molecular docking studies. Molecular docking of both components was done with aquaporin and arginase. Cell membrane integrity in bacteria is an important factor for the viability of the cell. In particular, cell membrane permeability has an important place in the regulation of intracellular and extracellular osmotic pressure. Aqauporins are important pumps in the cell membrane and are target molecules of many bacteriocidal agents. Therefore, molecular docking with aquaporin and major components of extract was investigated. Another protein (enzyme) arginase was investigated within the scope of molecular docking. Arginase is an enzyme involved in the metabolism of arginine, which is an important pathway in bacterial pathogenesis. Inhibitions in this pathway in bacterial infections are an important beacon of hope for treatment. For this reason, the interaction of the major components in the extract with arginase was also investigated. The results of molecular docking based on binding energy revealed that phytol and gallic acid have capable to interact with aquaporin-z and arginase. The phytol made hydrogen bonding and hydrophobic interaction to amino acid residues of aquaporin-z with a binding energy of − 5.21 kcal/mol and inhibition constant of 150.52 uM (Fig. 4a, Table 2). The amino acid residues of aquaporin-z have interacted with gallic acid made hydrogen bond and hydrophobic interactions which involved binding energy of − 3.62 kcal/mol and inhibition constant of 2.21 mM (Fig. 4b, Table 2). With binding energy of − 3.75 kcal/mol and inhibition constant of 1.78 mM, the phytol formed hydrogen bonds and hydrophobic interactions with amino acid residues of arginase (Fig. 5a, Table 2). Gallic acid formed hydrogen bonds and hydrophobic interactions with arginase amino acid residues with a binding energy of − 4.13 kcal/mol and an inhibition constant of 943.01 uM (Fig. 5b, Table 2). Phytol and gallic acid interact with many proteins and provide protection against various microbial infections. In literature, it is reported that phytol makes molecular docking by forming hydrogen bonds with glycine amino acids in the receptor to which *Mycobacterium tuberculosis* binds, and causes receptor inhibition^52^. Arsianti et al.^53^ reported that gallic acid derivatives interact strongly with malarial dihydrofolate reductase in molecular docking study and reported that gallic acid derivatives were protective against malaria caused by *Plasmodium parasite*.

**Table 2:** Binding affinities and potential molecular interactions of gallic acid and phytol with aquaporin-z and arginase.

		Free energy of binding (kcal/mol)	Inhibition constant (Ki)	Hydrogen bond interactions	Hydrophobic interactions
Aquaporin-z	Phytol	− 5.21	150.52 uM	GLY28 LEU25 VAL24	ALA23 (× 3) ALA27 ALA117 (× 2) ALA117 PRO126 VAL24 TRP200)
	Gallic acid	− 3.62	2.21 mM	SER114 PHE116 ALA117 VAL24 ALA23 SER114	VAL24
Arginase	Phytol	− 3.75	1.78 mM	MET203 GLU250	HIS252 (× 4) ARG249 (× 4) LEU253 (× 2) LEU298 ARG249
	Gallic acid	− 4.13	943.01 uM	MET203 (× 2) ARG249 (× 2) GLU250 THR204	LEU253

### Anti-proliferative activity

The anti-proliferative activity of *A. lividus* extract was determined by the *Allium* test based on MI percentages and the results are given in Table 3. The anti-proliferative effect of *A. lividus* extract was evaluated by comparing the negative and positive control groups. While 291 out of 5.000 cells in the negative control group were in the dividing stage, the number of dividing cells decreased by 2.9 times to 99 in the glyphosate treated group. The proliferation-reducing effect of glyphosate is associated with abnormality in cell cycle checkpoints, particularly at the G_2_/M transition point^54,55^. *A. lividus* extract showed a proliferation-reducing effect in dividing *A. cepa* meristem cells. It has been determined that *A. lividus* extract has a reducing effect on the number of dividing cells and exhibits an anti-proliferative effect of 25.7% compared to the control group. These results indicate that *A. lividus* has an anti-proliferative effect, but does not cause a toxic effect as far as glyphosate.

**Table 3:** Anti-proliferative activity of *A. lividus* extract.

	Number of dividing cells*	Number of cells in interphase	MI (%)
Negative control	291	4709	5.82
Glyphosate	99	4901	1.98
*A*. *lividus* extract	216	4814	4.32

*Indicates the number of cells in prophase, metaphase, anaphase and telophase.

The anti-proliferative effect of *A. lividus* extract can be explained by the active ingredients it contains. Tannins and flavonoids can delay or reduce cell division and inhibit cell division in *A. cepa*^56^. Many plant sterols, such as β-sitosterol, also have a retarding effect on cell division. Lopez-Garcia et al.^57^ reported that high sterols such as β-sitosterol, campesterol and stigmasterol obtained from plants have anti-proliferative effects. β-sitosterol, which was detected in the GC–MS analysis of *A. lividus* extract, contributes to the anti-proliferative effect. Gallic acid, the major compound detected in *A. lividus* extract, has been found to exhibit dose-dependent increasing anti-proliferative activity as well as anti-oxidant activity^58^. The fact that many agents obtained from natural sources and used in cancer treatment act by inhibiting the proliferation of cancer cells, reveals the importance of researching plants with anti-proliferative activity such as *A. lividus*. In addition, the anti-proliferative capacity of *A. lividus* extract, which does not show mutagenic activity and exhibits anti-mutagenic activity, also reveals its anti-aging activity.

### Molecular docking for anti-proliferative activity

**Figure 6:**
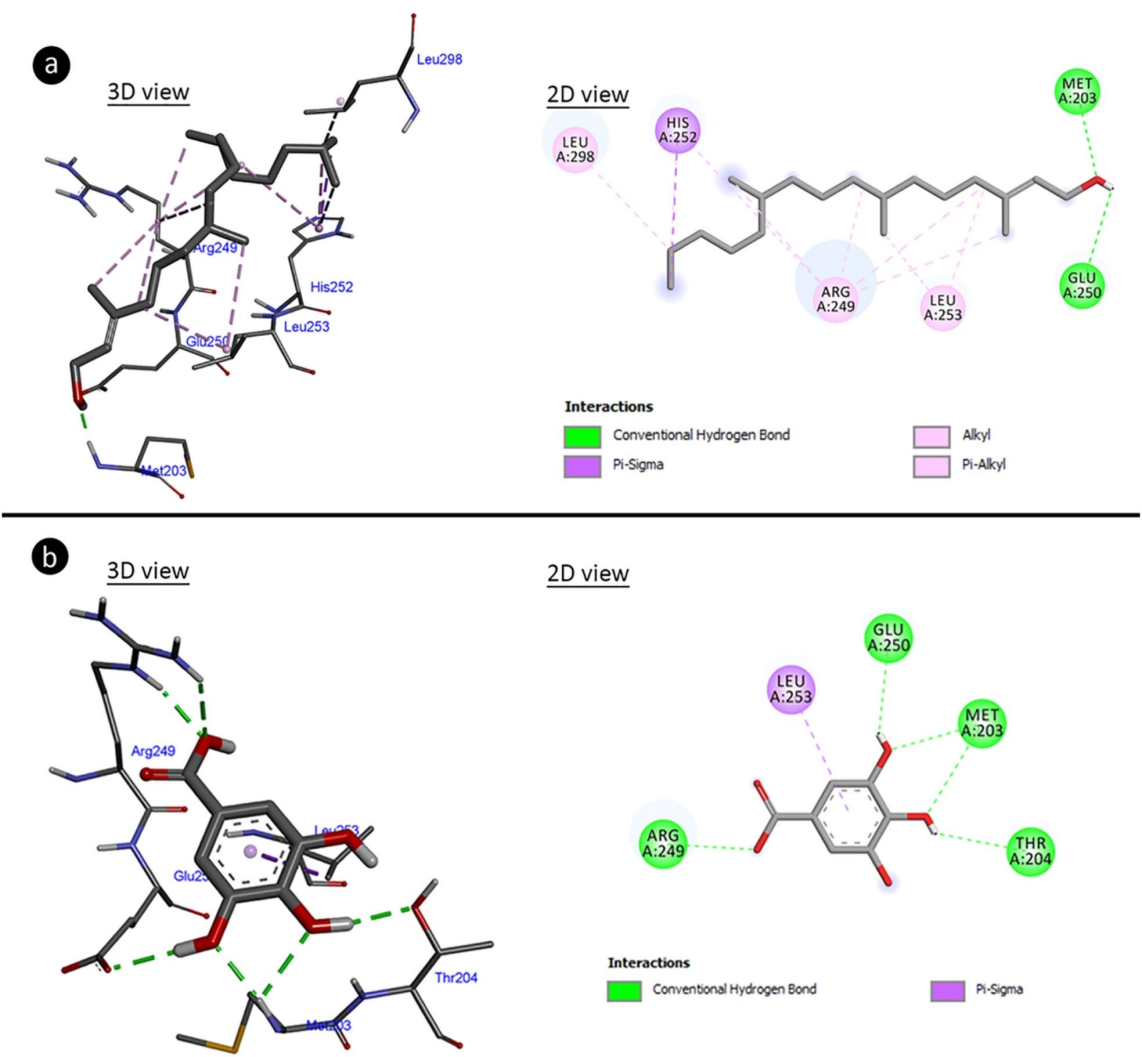
Molecular docking of gallic acid and phytol with telomerase. (**a**) Telomerase-phytol complex, (**b**) telomerase-gallic acid complex.

In order to evaluate the anti-proliferative effect mechanism of the extract, molecular docking of the major active compounds of the extract and telomerase enzyme was performed. Telomerase is the enzyme that prevents the shortening of telomeres at the ends of chromosomes, and telomeres in plants are protected by telomerase, as in mammals. There is intense telomerase activity especially in highly dividing meristem cells such as flowers and flower buds, seedlings, young and middle-aged leaves and root tips^59^. The decrease in telomerase activity causes regression in cell proliferation. For this reason, the interaction of gallic acid and phytol, which are the major components of *A.lividus* extract, with telomerase was investigated. The phytol made hydrogen bonding and hydrophobic interaction to amino acid residues of telomerase with binding energy − 6.25 kcal/mol and inhibition constant 26.01 uM (Fig. 6a, Table 4). The amino acid residues of telomerase have interacted with gallic acid made hydrogen bond and hydrophobic interactions which involved binding energy − 5.02 kcal/mol and inhibition constant 207.95 uM (Fig. 6b, Table 4). Regulation of telomere length and telomerase inhibition is defined as an important strategy in cancer diagnosis and treatment. In the literature, molecular docking of catechin with telomerase, which is frequently found in plants, was investigated and it has been reported that catechin bound to N-terminal amino acids has a potential anti-cancer effect. The interaction of gallic acid and phytol with telomerase demonstrated by molecular docking shows that *A. lividus* containing these components has a high anti-proliferative activity and naturally anti-cancer effect^60^.

**Table 4:** Binding affinities and potential molecular interactions of gallic acid and phytol with telomerase.

		Free energy of binding (kcal/mol)	Inhibition constant (Ki) (uM)	Hydrogen bond interactions	Hydrophobic interactions
Telomerase	Phytol	− 6.25	26.0	ARG486 (× 2)	MET482 (× 3) ILE497 (× 2) LEU554 (× 3) ILE550 MET483 ARG486 PHE478 PHE494 (× 4) TYR551 (× 3)
	Gallic acid	− 5.02	207.95	ARG486 (× 2)	ILE550 (× 2) ILE550 PHE494

### Anti-mutagenic activity

**Figure 7:**
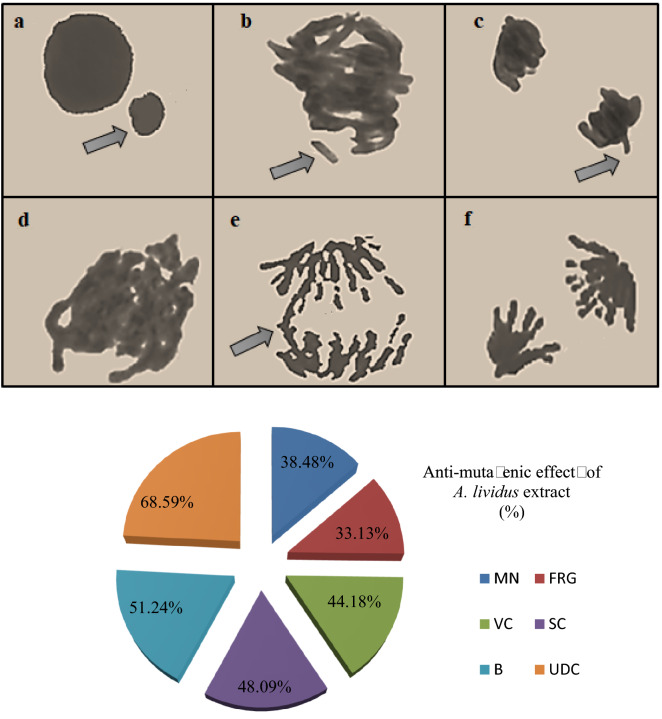
Genotoxicity induced by NaN_3_ and anti-mutagenic effects of *A. lividus* extract. (**a**) MN, (**b**) fragment, (c) vagrant chromosome, (**d**) sticky chromosome, (**e**) bridge, (**f**) unequal distribution of chromatin.

The anti-mutagenic activity of *A. lividus* extract was determined using the *Allium* test and the results are given in Table 5. As seen in the table, no statistically significant differences were observed between Groups I and III (p > 0.05). This result indicates that *A. lividus* extract does not cause any mutagenic effect, formation of CAs or MN. Serious mutagenic effects were detected in the positive control group. High frequency of MN formation, fragments, sticky chromosomes, bridges, vagrant chromosomes and unequal distribution of chromatin were detected in the NaN_3_ treated group (Fig. 7). NaN_3_ exhibits a mutagenic effect by causing chromosomal breaks and deficiencies in the DNA repair mechanism^61^. Chromosomal abnormalities such as fragments, bridges, vagrant chromosomes and unequal distribution of chromatin indicate aneugenic and clastogenic effects^62,63^. Abnormalities detected in the positive control group in this study confirmed the mutagenic effect of NaN_3_. In Group IV, in which NaN_3_+ extract was applied, there was a decrease in the frequency of these abnormalities. MN frequency decreased by 1.1 times in Group IV compared to the positive control group. This result revealed that the plant extract has a strong protective property and the frequency of NaN_3_-induced MN decreased.

**Table 5:** Frequency of MN and CAs frequency in experimental groups.

Damages	Group I	Group II	Group III	Group IV
MN	0.60 ± 0.52^d^	70.50 ± 8.10^a^	0.32 ± 0.12^d^	43.60 ± 8.62^c^
FRG	0.00 ± 0.00^d^	67.30 ± 8.91^a^	0.00 ± 0.00^d^	45.00 ± 9.58^c^
VC	0.00 ± 0.00^d^	50.70 ± 8.62^a^	0.00 ± 0.00^d^	28.30 ± 8.78^c^
SC	0.00 ± 0.00^d^	36.80 ± 9.02^a^	0.00 ± 0.00^d^	19.10 ± 6.82^c^
B	0.00 ± 0.00^d^	28.10 ± 8.16^a^	0.00 ± 0.00^d^	13.70 ± 5.81^c^
UDC	0.00 ± 0.00^d^	20.70 ± 6.00^a^	0.00 ± 0.00^d^	6.50 ± 3.10^c^

*MN* micronucleus, *FRG* fragment, *VC* vagrant chromosome, *SC* sticky chromosome, *B* bridge, *UDC* unequal distribution of chromatin.

1.000 cells were counted in each group for MN and CAs. Means shown with different letters^(a–d)^ on the same line are statistically significant (p < 0.05).

Similarly, the frequency of CAs decreased in the extract applied group and *A. lividus* extract exhibited an anti-mutagenic effect in the range of 33.13–68.59% against NaN_3_-induced chromosomal damages. The highest anti-mutagenic effect was determined as 68.59% against unequal distribution of chromatin. *A. lividus* extract, which provides 33.13%, 48.09%, 51.24% and 44.18% reductions in the fragment, sticky chromosome, bridge and vagrant chromosome frequencies, respectively, exhibited anti-mutagenic effects at different rates (Fig. 7). The reducing effect of *A. lividus* extract against CAs and MN formations is related to the active ingredients it contains. Phytol, one of the major compounds detected in the content, is an important factor in the emergence of the anti-mutagenic effect. Phytol exhibits anti-mutagenic activity by activating enzymatic detoxification systems, capturing radicals produced by mutagens, preventing DNA damage and scavenging radicals^64^. Gallic acid, another major compound detected in the extract, acts as a nucleophile that clears electrophilic mutagens. It also prevents the transfer of mutagens to the cytosol by binding to their carriers in the cell membrane^65^. It is reported in the literature that gallic acid, which reduces NaN_3_ mutagenicity by 82%, is one of the most effective anti-mutagenic phenols^66^. Although there is no study in the literature evaluating the anti-mutagenic effect of *A. lividus* extract, the anti-mutagenic activities of different *Amaranthus* species have been investigated by various tests. Kumari et al.^67^ found that 100 μg/mL *A. viridis* extract significantly reduced H_2_O_2_-induced DNA damage. Jadhav and Biradar^68^ reported that the mutagenic effect induced by ethyl methane sulfonate was inhibited by 96% with *Amaranthus hybridus* extract and 95% by *Amaranthus spinosus* extract.

## Conclusion

Mutagenic and carcinogenic compounds that we are exposed to in daily life cause serious toxic effects in organisms. To reduce or eliminate these toxic effects, exposure should be avoided. However, the increasing industrialization and the frequent use of chemicals in many industrial sectors make this exposure unavoidable. In cases where exposure cannot be avoided, natural compounds with important biological effects such as anti-microbial, anti-oxidant and anti-mutagenic should be consumed in the daily diet in order to reduce the toxic effects. For this reason, researching the biological and pharmacological effects of natural plant sources consumed as food comes to the fore. In the literature, there are many studies investigating the phytochemical contents and biological functions of plant extracts. However, the rich species diversity of plant species in the world and Turkey makes these studies insufficient. Plant extracts obtained from natural sources have been used in the treatment of many diseases for many years. Especially increasing cancer cases reveal the importance of researching plants with anti-mutagenic and anti-proliferative activities that inhibit the proliferation of cancer cells. In this study, the anti-mutagenic, anti-oxidant, anti-microbial and anti-proliferative effects of *A. lividus* extract, which is consumed as food in Turkey and many countries, were investigated. Phytochemical analysis of the extract content was also investigated and biological effects were associated with these ingredients. The presence of active ingredients such as gallic acid, phytol, caffeic acid, quercetin and vanillin was determined in the extract. A. lividus extract, which has intense active content, has superoxide and DPPH scavenging activity, strong antimicrobial and antifungal activity, anti-mutagenic and anti-proliferative activity. The mechanism of anti-bacterial and anti-proliferative activity was elucidated by molecular docking. Gallic acid and phytol, which are the major components in the extract, interact with aquaporin and arginase in bacteria, creating an antibacterial effect by toxic effect on bacterial viability. Gallic acid and phytol also interacted with the telomerase enzyme, which has a cell division-enhancing effect, causing deterioration in the enzyme structure and anti-proliferative effect. As a result, consumption of *A. lividus* in the daily diet with its strong biological properties will provide protection against many diseases and this study will be a guide for the research of natural resources (Suppl. Table 1).

## Supplementary Information

Supplementary Tables.
