# Supplementary material for: Molecular docking assisted biological functions and phytochemical screening of Amaranthus lividus L. extract
Source: Sci Rep. 2022 Mar 12;12:4308. doi: 10.1038/s41598-022-08421-8 (PMC8918320; doi:10.1038/s41598-022-08421-8)
Supplement: Supplementary file 1 — Supplementary Tables. [file 41598_2022_8421_MOESM1_ESM.docx]

**Table 1.** Drug-likeness properties of phytol and gallic acid

| **Compound** | **LogP** | **TPSA** | **natoms** | **MW** | **nON** | **nOHNH** | **nviol** | **nrotb** | **volume** |
| --- | --- | --- | --- | --- | --- | --- | --- | --- | --- |
| phytol | 6.76 | 20.23 | 21 | 296.54 | 1 | 1 | 1 | 13 | 349.38 |
| gallic acid | 0.59 | 97.98 | 12 | 170.12 | 5 | 4 | 0 | 1 | 135.10 |

TPSA: Total polar surface area (drug transport properties), natoms: Number of atoms, MW: Molecular weight (g/mol), nON: Number of hydrogen bond acceptors, nOHHN: Number of hydrogen bond donors, nviol: Number of Lipinski’s rule of five parameters violations, nrotb: Number of Rotatable Bonds (molecular flexibility).

**Table 2.** Biological activities of phytol and gallic acid

| **Compound** | **GPRC L** | **ICM** | **KI** | **NRL** | **PI** | **EI** |
| --- | --- | --- | --- | --- | --- | --- |
| phytol | 0.11 | 0.16 | -0.32 | 0.35 | 0.00 | 0.31 |
| gallic acid | -0.77 | -0.26 | -0.88 | -0.52 | -0.94 | -0.17 |

GPCR L: G-protein-coupled receptor ligands, ICM: ion channel modulator, KI: kinase inhibitor, NRL: nuclear receptor ligand, PI: protease inhibitor, EI: enzyme inhibitor.

Lipinski's rule of five states that an orally active drug should have no more than 10 hydrogen bond acceptors (particularly N and O groups), no more than 5 hydrogen bond donors (OH and NH groups), a partition coefficient log P of less than 5, a molecular weight of less than 500 g/mol, and no more than 4 violations. Gallic acid complied with all the lipinski rules, while phytol did not comply with the log P<5 rule, but met all the other rules. The total of particle additives is used to determine Molecular Polar Surface Area (TPSA). Polar pieces containing O and N centers are taken into account. PSA has been shown to be a powerful drug-related descriptor, including intestine absorption, blood-brain barrier penetration, and bioavailability. Molecules having a PSA value less than 140 are likely to absorb better in the intestine. According to the data in Table 1, phytol and gallic acid absorption from the gut is predicted to be good.

Table 2 shows the biological activity of phytol and gallic acid as G-protein-coupled receptor (GPCR) ligands, ion channel modulators, nuclear receptor ligands, kinase and protease enzyme inhibitors. The possibility of bioactivity for organic compounds, bioactivity value is higher than 0.00 for active, between -0.50 and 0.0 for moderately active, and less than -0.50 for inactive. The data showed that phytol is active as a G-protein-coupled receptor ligand, ion channel modulator, nuclear receptor ligand and enzyme inhibitor, but moderately active as a kinase inhibitor. Gallic acid was determined to be inactive as a G-protein-coupled receptor ligand, kinase inhibitor, nuclear receptor ligand and protease inhibitor, and moderately active as an ion channel modulator.
